# Discourses leading to diagnostic excess: an ethnopsychological study in Italian schools

**DOI:** 10.3389/fpsyg.2025.1672345

**Published:** 2026-01-12

**Authors:** Antonio Iudici, Cristiana Caraglia, Paolo Cottone

**Affiliations:** 1 Department of Philosophy, Sociology, Education and Applied Psychology of Padua (FISPPA), University of Padua, Padua, Italy; 2 Institute of Psychology and Psychotherapy, Padova and Milano, Milan, Italy

**Keywords:** communication, diagnosis, disorder, school, special educational needs

## Abstract

The increase in requests for certifications has created uncertainty and changes in the way minors are perceived in the school environment. The way in which schools categorize minors can influence their developmental path, facilitating or compromising their psychological and sometimes even clinical development. This article explores how teachers, parents, and child neuropsychiatry services in Italy identify and communicate a student's difficulty that may lead to a diagnostic certification of special educational needs (SEN). Drawing on an ethnopsychological perspective and employing semi-structured interviews with 46 participants, the study investigates the discursive and interactional dynamics underlying referral practices in schools. Using Critical Discourse Analysis (CDA), we examine how perceptions of nonconformity with educational norms become grounds for suspecting pathology, and how such perceptions are shaped by institutional expectations, professional uncertainty, and the search for legitimacy. The results reveal three specific conceptualizations of children: the needy child, the demanding child, and the child with strengths and weaknesses. Findings reveal that difficulties are often framed through comparisons with peers, failure to meet didactic standards, and behavioral non-adherence to school rules. Certification is frequently requested as a strategy for managing uncertainty or delegating responsibility to specialists. The results highlight a systemic inclination toward medicalising school diversity, exacerbated by communication gaps between schools, families, and health services. The study recommends clearer protocols, enhanced teacher training, and culturally sensitive approaches to promote inclusive and dialogic educational practices.

## 1 Introduction

The increase in requests for psychological and psychiatric certifications for children with special educational needs is a complex phenomenon that deserves careful analysis. In recent years, there has been growing concern about the current excess of certifications for minors (Moynihan et al., 2012), due to a complex interaction of social, educational, and cultural factors. While in some cases certifications are necessary and beneficial, it is important to be aware of the risks of diagnostic excess and at the same time understand if there are additional reasons that push schools and teachers to request certifications. Numerous authors discuss how the general tendency of society to medicalize behavioral and learning problems can also influence the assessment of minors, contributing to the increase in diagnoses (Poma, 2008; Frances, 2013; Conrad and Bergey, 2014; Moynihan et al., 2012).

For some authors (Swann et al., 2005), attention to child treatment is due to the idea that early intervention means having a positive impact on the course of a long-term disorder. Other scholars are concerned about the costs and risks of excessive treatment when administered to minors who do not really need it (Conrad, 2007; Frances, 2010).

Both these schools of thought presuppose and share the fact that there is an increase in the use of nosographic diagnoses that is not always justified. Regardless of positions, the literature has questioned the reasons that have led to overdiagnosis, defined as diagnosing an anomaly not associated with a danger to people's health (Welch et al., 2012; Chiolero et al., 2013; Iannotti and Wang, 2013), which have no benefit from it (Moynihan et al., 2012) and may instead suffer psychological and economic damage (Welch, 2011). Making counterproductive or iatrogenic diagnoses has various ethical implications, especially toward minors (Hofmann, 2016; Rogers et al., 2019).

Among the areas that are most affected by the risk of excessive medicalization (Batstra et al., 2012; Sayal et al., 2018) we find the school, where the number of certifications has increased strongly in various parts of the world (Atladottir et al., 2015; Collishaw, 2015; Polanczyk et al., 2015). The data raises even greater concern when it involves minors, creating incomprehensible differences: for example, childhood neuropsychiatric diagnoses in the US have reached much higher percentage peaks than African and European ones, particularly for ADHD (Attention Deficit Hyperactivity Disorder), autism spectrum disorders, and bipolar disorders (Moreno et al., 2007; Boyle et al., 2011; Visser et al., 2014; Zablotsky et al., 2015). All this raises several doubts from an epidemiological point of view (Poma, 2008; Merikangas et al., 2009; Gnaulati, 2013).

One of the main drivers of this trend is the growing pressure for school performance. As highlighted by (Brinkman et al. 2009), the emphasis on results can push teachers to seek medical explanations for students' difficulties, leading to an increase in certification requests. Paradoxically, even greater awareness of learning disorders can contribute to this phenomenon. (Gibbs and Elliott 2015) suggest that a deeper knowledge of these disorders can lead to a tendency to “pathologize” behaviors that could fall within the normal variability of development.

Limited resources in classrooms also play a significant role. (Frances and Batstra 2013) note that the lack of means to manage large classes can push teachers to seek support through certifications, in the hope of obtaining additional assistance. Moreover, in some school systems, funding policies can create indirect incentives for increased diagnoses, as schools might receive additional funds for certified students (Tomlinson, 2012).

Social and parental expectations further contribute to this dynamic. (Stella 2004) emphasizes how often the reports that the school makes are also influenced by parents' reactions. Teacher training, as noted by (Pijl 2010), often emphasizes early identification of problems, but might not provide sufficient tools to manage diversity in the classroom without resorting to certifications. Today, middle social classes and aspiring parents increasingly demand certification to receive funding and resources for children who would otherwise struggle to achieve results in competitive and market-oriented school systems (Malacrida, 2004; Harwood, 2006; Armstrong, 2017). (Graham 2008) observes that growing parental expectations regarding children's school success can exert pressure on teachers to identify and “solve” any perceived difficulty.

Contributing to all this are also some needs of teachers, encouraged or forced to “raise standards” to be free from problematic and disturbing students (Singh, 2004; Blum, 2007; Rafalovich, 2013; Florian and Rouse, 2009; Tomlinson, 2012).

It is therefore of undoubted interest to understand what supports this process, and to do so it is necessary to understand how reporting typically occurs, that is, what criteria teachers use in requesting certification. At the international level, different approaches are observed. In the United Kingdom, (Gwernan-Jones and Burden 2010) describe an approach based on systematic observation and data collection, which includes performance monitoring, observation of classroom behavior, and comparison with peers. In the United States, the Response to Intervention (RTI) model, described by (Fuchs and Fuchs 2006), provides for gradual interventions before proceeding to a formal report. (Karande et al. 2007) note that in India many parents initially resist the idea, often due to social stigma, and this also limits teachers' reports. On the contrary, (Poon-McBrayer and Wong 2013) find that in Hong Kong many parents are proactive in seeking evaluations, which translates as a form of pressure on the school.

In the Italian context, there is no clear and shared practice in requesting certifications, although the Italian educational system was one of the first systems in Europe to promote an inclusive approach, which globally values the student's needs and differences, the latter considered a resource and a stimulus for learning (Dovigo and Ianes, 2008; Dovigo, 2017; Anastasiou et al., 2015). Some authors argue that the inclusive approach is inversely proportional to the very definition of special needs (Thomas and Loxley, 2022). Despite this, the number of certifications remains clearly increasing (Autorità Garante per l'Infanzia e l'Adolescenza, 2022; Lattke et al., 2022). In fact, regarding certifications related to Disability, in the last 20 years, the increase has been over 50%. Regarding Specific Learning Disorders (SLD), it went from 0.7% in the 2010/2011 school year to 3.2% in the 2017/2018 school year, con un aumento del 357.14% (Miur, 2019). The Istituto Superiore di Sanità (ISS) found that the prevalence of autism spectrum disorders increased from 1 in 150 children in 2000 to about 1 in 77 in 2019 [Istituto Superiore di Sanità, 2021]. For these reasons, our research aimed to explore the possible cultural discourses that can today lead schools to exceed in the request for psychological and psychiatric certifications in reference to minors with special educational needs.

Therefore, this research arises from the following research questions: How do teachers and parents notice a difficulty? How does participation and communication between teachers, parents, and neuropsychiatrists take place with regard to students with SEN? In particular, the purpose guiding this article is to understand the way in which, through a transversal reading of the use of discourses, teachers, parents, and neuropsychiatrists identify a difficulty and communicate the difficulty of a presumed SEN minor, comparing interventions and regulations proposed by European educational systems. What leads to increasingly requesting diagnostic evaluations?

The proposal was to detect which discourses mediate the request for certification and how the subsequent evaluation is used by the various roles involved. The aim was to acquire research data that can allow a more conscious use of diagnostic evaluations, in favor of inclusive interactions.

## 2 Method

### 2.1 Framework

The theoretical perspective guiding this study is the Interactionist Perspective (Salvini, 1998; Iudici et al., 2022), within which knowledge is the result of a construction of meanings shared by different subjects interacting with each other. These subjects, belonging to the same sociocultural community, construct and transmit, through language, their dominant cultural values, which can orient a specific identity conduct. To understand the experiences, needs, and meanings that individuals attribute to a process of detection and communication of a difficulty experienced by a presumed SEN minor and the way in which they generate pragmatic repercussions from the relationships established in the context in which they act, we chose to resort to qualitative research, which studies phenomena in the complexity of their natural contexts (Denzin and Lincoln, 2005; Flick, 2009).

### 2.2 Deconstructing diversity: conceptual elements in the current scientific debate

The conceptualization and management of diversity in schools remain controversial issues in the field of education (Tassinari Rogalin et al., 2024; Bassi et al., 2024). Our work focuses on the processes through which diversity is constructed, categorized, and addressed in the school context, with particular attention to diagnostic excess. In reference to the current debate, some specific conceptualizations support the analysis and discussion of these processes.

Firstly, the presence-participation-achievement (PPA) model, developed by (Ainscow and Miles 2009), which is articulated in three-dimensional framework. “Presence” refers to physical access to education for all students, regardless of their characteristics. “Participation” concerns the quality of the educational experience, emphasizing the active involvement of students in the learning process and school life. “Achievement” goes beyond academic results, including personal and social development. The application of the model requires continuous monitoring of these three dimensions, implementing targeted strategies to improve each aspect. The advantages include a holistic vision of inclusion and a practical framework to guide educational policies and practices. The effectiveness of the model depends on its application and contextualized interpretation by those who practice it; indeed, it is not always easy to adapt it to specific schools. This framework provides a useful structure for setting up inclusive activities, although the three dimensions may risk oversimplifying the complexity of the educational experience, schools, and students. This approach does not consider systemic barriers to inclusion, focusing mainly on the individual aspects of the psychological experience within schooling. As some authors have argued, the risk is to indirectly perpetuate a deficit approach, focusing on students' “lacks” rather than on the inadequacies of the educational system itself (Slee, 2013).

Moreover, the tendency to interpret individual differences, as already seen in the introduction of this work, through a medicalized lens has led to a significant increase in diagnoses and clinical interventions in the educational context, a phenomenon that (Conrad 2007) defines as the medicalization of diversity. This approach, although apparently objective and scientific, raises several critical issues that deserve careful consideration. Firstly, there is a concrete risk of reductionism, where human complexity is reduced to simplified diagnostic categories, losing sight of each individual's uniqueness. Furthermore, this approach tends to shift responsibility from social and environmental barriers to presumed individual problems and “deficiencies,” somewhat deresponsibilizing the school system. Indeed, the focus on individualized interventions based on medical diagnoses can divert attention from the systemic changes necessary to create a truly inclusive environment (Lauchlan and Boyle, 2007; Haug, 2016).

As is well known, diagnostic labels, while sometimes useful for accessing specific supports that would otherwise not be available, can also lead to stereotypes and discrimination, negatively influencing students' self-perception and educators' expectations (Shifrer, 2013; Barg et al., 2010; Gibbs and Elliott, 2015). Also in response to these issues, some socio-critical perspectives, such as those proposed by (Annamma et al. 2013) and (Artiles et al. 2011), offer an alternative to the medicalized view, focusing on the social construction of difference. This new framework allows us to critically evaluate how discursive and institutional practices in schools contribute to creating and maintaining categories of “normality” and “diversity” (Neri et al., 2020).

On the other hand, this approach also presents some challenges, especially in its practical application. The complexity of implementing these theoretical ideas into concrete educational practices can be considerable, requiring a fundamental rethinking of existing school structures and practices. Moreover, challenging existing power structures can encounter significant resistance from institutions and individuals accustomed to operating within more traditional paradigms. Another limitation is the risk of placing excessive emphasis on the social construction of difference, potentially underestimating the objective challenges that individuals must face, requiring a delicate balance between recognizing social influences and attending to individual needs.

The critical analysis of these three approaches reveals the complexity and contradictions in the processes of constructing diversity in schools. The need for a more nuanced and integrated approach clearly emerges that can recognize the limitations of the PPS model while utilizing its useful aspects, challenge excessive medicalization without denying the value of specialized supports when necessary, and integrate socio-critical perspectives while maintaining a pragmatic focus on students' individual needs.

### 2.3 Aims

To promote theoretical, methodological, and operational reflections inherent to the practices that characterize the detection and communication of difficulties, two objectives were outlined:

1. Explore how different roles identify a difficulty/configure a special educational need
2. Explore how different roles communicate the outcome of the difficulty in order to report a difficulty

### 2.4 Participants, data collection and sampling method

The participants identified for this research work are 46: 28 primary and secondary school teachers, including eight males and 20 females; nine parents of minors with special educational needs; nine operators from the Child Neuropsychiatry Service, of which two are psychologists and seven are neuropsychiatrists. Participants were involved by contacting principals and referents of two schools and two neuropsychiatry services in Veneto and Lombardy. We wrote to them to ask for their availability to participate in the research, sending them some research presentation documents. They then proposed the research to teachers and parents of the schools they directed, who then contacted the research authors. The research was also proposed to some Services for developmental age that collaborate with schools in the regions of Lombardy (Operational Unit of Neuropsychiatry for Childhood and Adolescence, UONPIA) and Veneto (Services for Adolescence). Some participants were recruited through other participants, who were asked to refer potential research participants, thus expanding the sample through their social networks.

The sampling method used in this research can be defined as non-probabilistic sampling, more specifically a purposive sampling (Etikan et al., 2016) combined with snowball sampling (Robinson, 2014; Palinkas et al., 2015) (Table 1). The purposive sampling was identified among (a) educational institutions that could host minors of different ages and have different levels: primary, lower and upper secondary schools; specifically teacher and parents (Tables 2, 3) (b) psychological and neuropsychological services for adolescents that collaborated with educational institutions (Table 4). This method allowed researchers to reach specific groups (teachers, parents, healthcare and educational operators) directly involved with students with special educational needs. The invited schools were identified within a range of about 300 km from the University of Padua; the schools where the research was conducted responded spontaneously. Once initial contacts were established, recruitment extended through word-of-mouth within the school, with participants suggesting other potential participants. Indeed, snowball sampling is particularly effective for recruiting participants where collaboration networks between operators are already present and where participants belong to a more specific population, namely those dealing with social inclusion of SEN students.

**Table 1:** Participants.: Gender, School/service, Distrect, Years of experience.

**Participants number**	**Participants role**	**Gender**	**Scuola/Service**	**Distrect comunity**	**Experience (years)**
1	Teacher	F	Primary school	Lombardy region	15
2	Teacher	F	Primary school	Lombardy region	12
3	Teacher	F	Primary school	Lombardy region	17
4	Teacher	M	Primary school	Lombardy region	4
5	Teacher	F	Primary school	Lombardy region	22
6	Teacher	F	Primary school	Lombardy region	19
7	Teacher	F	Primary school	Lombardy region	20
8	Teacher	F	Primary school	Lombardy region	2
9	Teacher	M	Primary school	Lombardy region	6
10	Teacher	M	Primary school	Lombardy region	12
11	Teacher	F	Primary school	Lombardy region	10
12	Teacher	F	Primary school	Lombardy region	18
13	Teacher	F	Secondary school first grade	Lombardy region	8
14	Teacher	M	Secondary school first grade	Lombardy region	9
15	Teacher	F	Secondary school first grade	Lombardy region	21
16	Teacher	F	Secondary school first grade	Lombardy region	18
17	Teacher	F	Primary school	Veneto region	23
18	Teacher	F	Primary school	Veneto region	17
19	Teacher	F	Primary school	Veneto region	18
20	Teacher	F	Primary school	Veneto region	15
21	Teacher	M	Primary school	Veneto region	6
22	Teacher	F	Primary school	Veneto region	8
23	Teacher	M	Primary school	Veneto region	12
24	Teacher	F	Primary school	Veneto region	21
25	Teacher	F	Primary school	Veneto region	5
26	Teacher	F	Secondary school first grade	Veneto region	12
27	Teacher	F	Secondary school first grade	Veneto region	17
28	Teacher	M	Secondary school first grade	Veneto region	15
29	Child neuropsychiatrist	F	The Child Neuropsychiatry Service (UONPIA)	Lombardy region	18
30	Child neuropsychiatrist	F	The Child Neuropsychiatry Service (UONPIA)	Lombardy region	16
31	Psychologist	F	The Child Neuropsychiatry Service (UONPIA)	Lombardy region	12
32	Child neuropsychiatrist	F	The Child Neuropsychiatry Service (UONPIA)	Lombardy region	20
33	Child neuropsychiatrist	F	The Child Neuropsychiatry Service (UONPIA)	Lombardy region	19
34	Child neuropsychiatrist	F	Developmental age service	Veneto region	16
35	Psychologist	F	Developmental age service	Veneto region	20
36	Child neuropsychiatrist	F	Developmental age service	Veneto region	21
37	Child neuropsychiatrist	F	Developmental age service	Veneto region	18
38	Parent	M	Primary school	Lombardy region	-
39	Parent	F	Primary school	Lombardy region	-
40	Parent	F	Primary school	Lombardy region	-
41	Parent	F	Primary school	Lombardy region	-
42	Parent	F	Primary school	Lombardy region	-
43	Parent	F	Primary school	Veneto region	-
44	Parent	M	Primary school	Veneto region	-
45	Parent	F	Primary school	Veneto region	-
46	Parent	F	Primary school	Veneto region	-

**Table 2:** Teachers data.

**N. Teachers**	**Male**	**Female**	**Scuola**	**Regione**
12	4	8	Primary school	Lombardia
4	1	3	Secondary school first grade	Lombardia
9	2	7	Primary school	Veneto
3	1	2	Secondary school first grade	Veneto
28	8	20		
100%	28.57	71.42		

**Table 3:** Parents data.

**N. Parents**	**Male**	**Female**	**School**	**Region**
5	1	4	Primary school	Lombardia
4	1	3	Primary school	Veneto
9	2	7	22.22%	
100%	20%	80%	77.77%	

**Table 4:** Services data.

**N. Neuropsychiatrists**	**Male**	**Female**	**Service**	**Region**
5	0	5	The Child Neuropsychiatry Service (UONPIA)	Lombardia
4	0	4	Developmental age service	Veneto
9	0	9		
100%	0%	100%		

### 2.5 Investigation method: the semi-structured interview

To explore the perspectives of Teachers, Families, and Service Operators in relation to both research objectives, we opted for the use of semi-structured interviews (Groeben et al., 1988). This methodology was chosen for its ability to investigate in depth the opinions of participants and to examine the interactive dynamics, both at the content and participation level, that are presumed to exist between these groups. The semi-structured interview is distinguished by its flexibility: it offers a general frame of reference that ensures coverage of all aspects relevant to the research objectives, while allowing the interviewer to adapt to the natural flow of the conversation (Cohen and Crabtree, 2008). This method can be described as a guided conversation, conducted by the interviewer with selected individuals, primarily aimed at acquiring knowledge and exploring new perspectives (Corbetta, 1999; Harrell and Bradley, 2009). This approach allows us to maintain focus on the research objectives, adapt to the unique responses of each participant, explore any emerging themes not initially anticipated, and create adequate conditions to respond dialogically in an open and natural way. In this way, we aim to obtain a rich and nuanced understanding of the experiences and perceptions of the various actors involved in the educational process (Table 5).

**Table 5:** Research protocol.

**Interview questions**
1. What criteria do you use to describe the problems/difficulties of students that may lead to a diagnostic assessment?
2. What signs or symptoms do you consider worthy of clinical attention?
3. Is there a shared observation grid for making referrals?
4. How do teachers, parents and neuropsychiatry operators communicate with each other?
5. Are there network meetings?
6. How do they take place?

### 2.6 Data analysis and coding

To understand the process that guides the choice of criteria regarding the detection of difficulties among the various roles and at the same time the discourses that lead to the increase in certifications, the interdisciplinary methodology of data analysis called CDA was chosen. This system of analysis, pioneered by (Fairclough 1995), studies social phenomena based on the assumption that there is a dialectical relationship and mutual influence between language and the society in which it is used, as language is considered a form of action in the context of social relations (Fairclough, 2013; Fabbro, 2020). This system does not focus exclusively on the content exposed through language but provides tools to examine how such common-sense discourses are maintained and reproduced within specific social contexts (Limone, 2012a,b). Discourses are not individual property but have a collective function as they are produced by the intersection between people who use language and the cultural context of reference (Harré, 2016; Harré and van Langenhove, 1999). (Fairclough 2013) describes the three analysis processes linked to three dimensions of discourse that characterize the CDA model: text analysis, discursive practice, and finally, social analysis. In particular, during text analysis, the researcher describes how grammatical and lexical choices orient positioning toward the topic. Subsequently, during discursive practice, the researcher offers a summary interpretation of how activities and relationships present in the context are interpreted and meanings are attributed to them, without reporting the words actually used by the participants. Attention is paid to how language is used in the distinctive activities of a particular institution, to the dynamic relationships and multiple positions that subjects can assume within the situation, paying attention to the social origin of the resources on which the interpreter relies to interpret this point (Fairclough, 2003, 2013) (Table 6).

**Table 6:** Text analysis criteria.

**Steps**	**Conceptual phase**	**Criteria**
1	Transcription	- Accurate recording of the text (audio or written) - Detection of paralinguistic elements (pauses, intonation, emphasis) - Annotation of significant contextual elements
2	Coding	- In-depth and repeated reading of the text to familiarize with the content - Identification of relevant units of meaning - Assignment of initial codes to these units - Theoretical coding matrix according to the three levels of analysis: textual, discursive, and social
3	Category construction	- Grouping codes into broader categories. - Identifying recurring themes and discourse patterns. - Exploration of relationships between categories - Attribution of the text to levels of theoretical analysis: a) Textual: detailed linguistic analysis (lexicon, grammar, structure) b) Discursive: examination of the processes of production, distribution, and consumption of the text c) Social: analysis of broader sociocultural practices that influence the discourse
4	Intertextual and interdiscursive analysis	- Analysis of connections between the analyzed text and other texts - Identification of how different discourses intersect in the text
5	Critical interpretation	- Linking linguistic analysis to social and power structures - Exploration of how discourse reproduces or challenges social inequalities - Consideration of the ideological implications of the discourse
6	Presentation of results	- Use of textual examples to support interpretations - Discussion of theoretical and practical implications of the analysis

Finally, during social analysis, the researcher explains how discourses are legitimized and modified within specific socio-cultural contexts, through the transversality of different points of view between roles. This explanation phase represents discourse as part of a social process, studying the implications and effects that these discourses can have on social structures, modifying or supporting them, respectively, through normative or creative relationships. Therefore, it is important to focus on the constitutive properties of the society being studied to understand how power relations affect the positioning of different social actors. Thus, social analysis aims to unveil such ideologies and underlying assumptions present in discourses and understand how members' resources are formed and modified at the social level (Fairclough, 2013).

### 2.7 Verification of scientific results integrity

To strengthen the robustness of our investigation, we explicitly delineated the epistemological framework of the research team, illustrating the theoretical and conceptual foundations previously mentioned. Deep knowledge of the context was ensured through preliminary interaction with school principals, who were informed of the purposes and objectives of the investigation. The examination of the collected material was carried out by each team member independently, followed and supervised by a collective comparison phase (triangulation). This strategy allowed for a richer and more nuanced understanding of the textual contents. The robustness of the results was corroborated through a meticulous exposition of the data classification criteria, employing a matrix of questions and coding parameters related to positioning. This methodological approach ensures the possibility of replicating collection and analysis procedures. We also provided a detailed account of the participants' characteristics. The possibility of extending the study's conclusions was enhanced by the richness of the responses provided. Our analytical approach, focused on identifying discursive positioning, ensured a meticulous interpretation of the text, thus increasing the accuracy of the results. The credibility of the research was further consolidated during the data acquisition and collection phases, as well as in the examination of the correlation between raw data and conclusions. Throughout the entire investigation process, we developed specific protocols for each question posed. Researchers constantly monitored adherence to the method, highlighting any critical issues as potential limitations of the study. During the analysis, responses not pertinent to the objectives of the questions were excluded from the final evaluation. The analysis of the results involved reflection among the members on the interpretations of the data. Alternative interpretations were also considered in order to reinforce the interpretation most suited to the theoretical references adopted. The analysis was also related to the socio-cultural context of the schools involved.

## 3 Results

The results below involve all three roles, as per the discourse methodology. Participants' interventions have been reported in function of the general discourse. These interventions have been reported based on the role and are thus denominated: Teachers (T1-T28), Parents (P1-P9), Service of Neuropsychiatrist (S1-NS9). At the end of this paragraph, we have included some ethnographic notes.

### 3.1 Comparison with others as a discourse to determine difficulty

One of the criteria that teachers and parents use to identify a difficulty is the comparison between children of the same age, applied to various areas such as behavior, learning, and age-related expectations. Some teachers reported, “I see that that boy is very different from others, especially those his age” (T8), “Compared to the boys I've had, he had something different, I don't know how to explain it” (T19). Similarly, a mother says: “Having experience with the older child, I saw that there was something wrong” (P14), while another stated, “He does things that are not appropriate for his age” (P6).

Quotes T8, T19, P14, and P6 show how teachers and parents consistently use comparative discourse to identify difficulties. This reflects a deeply rooted social practice that defines “normality” through comparison, potentially marginalizing individual differences.

Expressions used by parents include: “It wasn't what I would have expected from a child” (P9); “He seemed like a doll” (P11); “He should have been and instead” (P2). Or, a teacher explains: “I see what the errors can be and if I don't see them in other kids, then I make the parallel” (T7).

Statements from P9, P11, and P2 reveal how age-related expectations are socially constructed and used as a yardstick. This discourse can contribute to creating and maintaining rigid developmental norms.

Another criterion used is the duration of the problem or difficulty: the longer the problem persists, the more pervasive it is. In particular, a parent explains: “So much so that I became alarmed and after seeing that it was a situation that persisted over time, I told myself that we needed to intervene” (P1). A parent said: “At first you wait a bit, you think of everything, you hope it will improve, but then over time you say: there's something wrong” (T8). Another teacher stated: “First we try to wait, to observe the child calmly, but then you see that others make progress and he just doesn't” (T11). A neuropsychiatrist said, “Parents and teachers are asked to wait a bit, but then if the situation doesn't change, then maybe there is a problem” (S4). Quotes P1, T8, T11, and S4 show how the temporal persistence of a problem is discursively constructed as an indicator of severity. This could reflect and reinforce a deterministic view of student difficulties.

A third criterion used is “agreement” with colleagues. Or there is a tendency to legitimize the work done in teams among colleagues, as a “saving” source to identify the child's difficulty more reliably, “Fortunately, colleagues agreed with me, otherwise I wouldn't have known what to do” (T22). There is talk of agreement rather than comparison, and this leads to thinking that the positioning of teachers is more linked to procedural responsibilities than to the construction of shared reasoning. In any case, this shared agreement increases the reliability of the reporting by them. A teacher said, “When I have a problem with a student, I talk about it with others, and if others support it, we talk about it with others in the class council, and so they tell me if they agree” (T5). If, on the other hand, the child has a difficulty only with one teacher, the teacher tends to judge themselves, as if they were not able to help the child achieve the objectives, feeling more responsible for generating the difficulty. In particular, teamwork is considered “saving” (T28) and “a hand from heaven” (T17) by some teachers, particularly to make the assessment of the situation more “reliable,” as can be seen from this teacher's discourse: “I often compare with other teachers… in my opinion it's a good practice for data collection that makes the thing more reliable” (T5). Quotes T22, T5, T28, and T17 highlight how the discourse of consensus among colleagues is used to legitimize individual concerns. This can reflect and reproduce power hierarchies within the school system. If the problem is found by everyone, automatically, it can also lead to creating the idea of a greater severity of the minor and therefore the creation of a team to discuss the need to start a process of reporting to services with families, in which the principal is also present, as a teacher explains: “If it's a discourse only in my subject, maybe the problem is mine (teacher) and not the boy's; if instead the problem is common to all, we think about it” (T4). When the school procrastinates or does not recognize the child's difficulty, parents “point out” to teachers the difficulties based on their own experience.

A fourth criterion concerns “intellectual evidence.” Some teachers say: “Concretely, when there are situations of intellectual discomfort, they are evident” (T8) or “A disorder is more easily recognizable than another if such disorder is evident, based on the logic used” (T3). Another teacher stated, “At a glance, you can tell if someone has a serious problem” (T7). Quotes T8, T3, and T7 reveal a discourse that privileges “evident” difficulties, potentially marginalizing less visible but equally significant problems.

### 3.2 The discourse of failure to respond to didactic requests as a criterion of difficulty

Parents and teachers adopt divergent approaches in attempting to address the uncertainty that emerges when confronted with the challenges experienced by the student. While both seek to develop new strategies, their methodologies and perspectives often prove to be contrasting.

Within the family context, new strategies are experimented with that give rise to a personalized path between parent and child; this path responds more to the child's needs and involves a remodulation of the behavior implemented by the child, for example, a mother explains: “Compared to school rules, he preferred to do something else, he wanted to leave the class, he was bored, he did a little then needed other stimuli, while at home involving him in another way according to his needs and not those of the school, then in the personalized relationship he was also able to better modulate his behavior” (P9). A child neuropsychiatrist argues, “I think teachers start only from some standards they have in mind, which, however, do not consider the neurodiversity of minors” (S6). Quote S6 introduces a critical discourse on “neurodiversity,” challenging the dominant discourse of standardization in schools. This reflects a broader discursive struggle in the field of inclusive education. Conversely, within the school context, the teacher values a possible difficulty based on how much the child responds or not to the expectations linked to school norms and school requirements indicated in ministerial programs, in fact in the interviews only partially appear other aspects, such as interests, relationships, and interactions that a person is able to build within the school context. In particular, a teacher specifies: “The pupil is reported when he does not reach the minimum requirements of reading, writing, and calculation required by school norms and if the difficulty persists despite the various strategies implemented” (T4). Another teacher says, “I can make an assessment with respect to the program and what is established that the kids should do in the current year and only with respect to that you realize that someone has some relevant difficulty.” The relationship between standards and results is also evident in the following response offered by a middle school teacher who somewhat resignedly said, “We report when the child clearly does not reach the required basic level” (T13).

Quote P9 shows an alternative discourse based on personalization and adaptation to the child's needs, in contrast with the rigid school discourse highlighted in T4 and T13. This reveals tensions between different social practices and educational ideologies. Moreover, some parents confirm the criteria used by some teachers to detect difficulty: “Already during the first elementary school, the teachers reported that he did not follow what they said, does not respect the instructions and the simplest tasks” (P2). In particular, parents establish a difference between the school's requests and the boy's needs and criticize teachers because, being the teacher considered an expert figure able to mediate with the child's needs, there is a risk of considering “pathological” a behavior for the lack of adherence to school rules. From this, it can be seen that the prevalent way in which one relates to the minor is frequently considered based on the sole role of a student who learns didactically, paying less attention to personal and relational aspects.

When noticing some difficulties, the teacher often modifies the strategies implemented in order to find a way to help the minor. This happens once again to allow the minor to acquire the didactic tools and to demonstrate that everything has been done before reporting, as per the Services' indications. The strategies put in place almost always concern activities of a cognitive order, such as personalized or small group explanation, or with dispensative or compensatory tools. A teacher said: “We try to have them do a different task, doing examples together first and we explain it to them many times, but then the result often doesn't change.” Some teachers have stated that this is the reason why a teacher often works with the individual minor deemed to be in difficulty outside the classroom, when there is co-presence between teachers. For example, a teacher says, “Often in the face of a difficulty, we try several times to repeat the task to the boy, we say take your time, we try to verify if he understood, but often the answer is the same” (T10). Quotes about teachers' strategies (e.g., T10) reveal a discourse of “sufficient proof” before reporting, which could reflect and reproduce bureaucratic practices and power relations in the educational system.

### 3.3 The discourse of discomfort as an effect of non-adherence to environmental requests

Tendentially, this category includes situations where the discomfort detected in the student is in relation to how much their behavior is not adequate to the school context.

Some interviewed teachers focus on aspects related to conduct, “Some children play tricks, react badly to things we say and often throw things” (T18), “With certain children we have a lot of difficulty, they get up and don't respect the minimum rules of coexistence, they fight with others, the other day a child threw a desk against a girl who didn't want to lend him her pen” (T6). A primary school teacher focuses on how the child's behavior is very difficult to understand, “For example, I happened to see children who didn't play with others during the break, they stayed alone or tried to chase someone who didn't want to play with him” (T13). Quote T13 reveals how behaviors that do not conform to social norms (such as playing alone) are discursively constructed as indicators of “discomfort” or “diversity,” potentially pathologizing the natural variability of child behavior. Substantially when a child does not respect the explicit and implicit requests of the school organization, the suspicion of some form of diversity connoted in terms of discomfort seems to open up. A primary school teacher says, “When we do group work, he still does his own thing, doesn't respect other classmates, he decides, doesn't compare, he wants to do what he has in mind” (T21). Quotes T18, T6, and T21 show how the discourse of “appropriate” conduct is used to define and categorize student behavior. This discourse can serve to legitimize practices of control and discipline within the school context.

A primary school teacher, when asked to know through which criteria the reporting took place, said: “You can see that he's not well, he can't sit still, he reacts, he behaves badly, obviously he doesn't learn anything like that” (T20). In this case, the minor's discomfort would be the cause of not respecting the request to sit still. The theme of sitting still at desks is recurrent among teachers' responses. A teacher said, “I often find myself in difficulty as I realize that some children can't sit still and do the things that others do, they are not… how to say… socialized to do it yet we have to ask them to, this is our task” (T14). Quotes T20 and T14 highlight how “knowing how to sit still” is discursively constructed as a key indicator of wellbeing and school adaptation. This discourse reflects and reproduces traditional educational practices that can marginalize different learning styles and behaviors.

Similar modalities are also used by parents in recognizing their child's difficulties. Initially, within the family context, they establish the reality of the existence of a problem based on the unexpected behavior that the child puts in place. A parent of a primary school child describes that “Even in kindergarten, the teachers reported that he doesn't stay still, doesn't respect the rules” (P2).

Parents in detecting the child's difficulty use some expressions that show a bit of resignation, for example, the mother of a primary school child said “it wasn't what I would have expected from a child of this age” (P8). Another parent uses the word “doll” to indicate a child who disappoints behavioral expectations, putting in place “things not appropriate for his age, which maybe you expect from someone a bit older” (P1). Parents' quotes (P8, P1) reveal how age-related behavioral expectations are discursively constructed and used as a yardstick. This can contribute to creating and maintaining rigid developmental norms. A child neuropsychiatrist argues that “it is very difficult to understand if the problem is in the boy or the problem is the classmates or the class. I think that sometimes we ask these children, regardless of the problem, what perhaps they cannot give us.”

### 3.4 The request for consultation (or delegation) to the expert to reduce the difficulty attributed to the child

From the research, it emerges that when some teachers and parents become aware that their student or child disconfirms the norms and behavioral expectations of society, they experience feelings of concern and sometimes guilt. A parent said, “I start to realize that maybe I should have been closer to my son, maybe I should have helped him a little earlier or had him seen by someone” (P7). A teacher with much concern said, “I always think about M., and I think if I really did everything possible, maybe I should have asked for help from someone earlier, maybe I wasn't able to understand him well” (T15). These experiences are often generated by the desire to provide help to the boy, as one feels more responsible for having contributed to generating such difficulty. Quotes P7 and T15 reveal a discourse of guilt and personal responsibility. This reflects a tendency to individualize educational problems, potentially obscuring broader systemic factors. Moreover, when the first interventions of teachers and parents do not generate any difference with respect to the reported problems and they fail to find a meeting point, one solution is to turn to the school psychologist, when present. This can allow the use of a recognized figure in the school context and appointed to work on difficulties. Sometimes the real request is to verify if there is a psychological or psychopathological reason that explains the school failure. From what we have found, parents and teachers share the theory of being able to help the boy only with the help of a specialist. It is important to note how the consultation requested from the expert is not considered by some teachers and parents as a tool to question their own way of acting but is intended as a facilitation to reduce the problem attributed to the minor as soon as possible. A teacher says, “I need to know if the disorder is serious or not, otherwise I don't know what to do, I feel blocked” (T18), while another argues, “It is necessary for the specialist to tell us what we need to do, I am not a psychologist or psychiatrist and therefore I don't know if what I decide to do is right or not” (T23). Quotes such as T18 and T23 construct the expert as a salvific figure, capable of solving problems that teachers feel unable to address. This discourse can reinforce hierarchies of knowledge and power in the educational system. And also, “If I don't know what the child has, I can't understand how to move, how to help him. And if my way was counterproductive?” (T11) The focus of the request in this case is always on the minor, who will receive a diagnosis or functional report that will serve to justify and explain the problem.

In fact, some teachers negatively judge colleagues who choose to report a student immediately without waiting for the third year and according to them seek “elsewhere” the resolution of the difficulty, in particular “leaving the word to the experts” (T5). Quote T5 reveals a critical discourse toward immediate delegation to experts, suggesting tensions within the teaching community regarding reporting practices. As one teacher reported: “for some colleagues it's easier to delegate and report, so others think about it; and this happens too often, as if the main interest is to have one more resource” (T13). Some neuropsychiatrists generalize the way in which teachers consider some difficulties to arrive at reporting, based on a thought that derives from the experience over the years of neuropsychiatry services. In particular, these neuropsychiatrists specify that teachers approach in two different ways when they choose whether or not to report a student: “they emphasize certain difficulties in the report by inserting within the form to be filled out the crosses (which indicate) on the severity a bit forced” (S3) or prefer not to report “as they hope that the child improves or not to upset the families” (S1). Quote S3 suggests that teachers may discursively manipulate the severity of students' difficulties, reflecting potential systemic or individual pressures to obtain support. Quotes from neuropsychiatrists (e.g., S1) construct a discourse of inadequacy in teacher training, potentially justifying greater authority of experts in the decision-making process.

Specifically, some neuropsychiatrists judge teachers as insistent toward families so that help can be received from the services and believe that, in recent years, teachers implicitly request the start of the process to obtain a functional diagnosis as a form of help to understand how to manage the student's difficulties but “such difficulties are found mainly by the teaching staff and not by the parents” (S5). In particular, a child neuropsychiatrist explains: “In these requests I believe there is a request for help from these teachers who at a certain point say they haven't understood if they can ask for the performance of a task or an activity to the child, if they can lower the grade because he didn't commit or because he can't do it” (S3).

Some neuropsychiatrists criticize the training that a teacher possesses regarding the compilation of the diagnostic form in which to note the student's difficulties, as can be seen in these discourses: “The parameters are a bit those linked to the teacher's knowledge with respect to the requirements that the child should have reached even after a strengthening of some areas […] I have the impression that teachers are not trained at all on this. We give very detailed lessons and I think teachers don't know how to read what we write” (S1); while other neuropsychiatrists explicitly state that they understand the position of teachers, in fact an interviewee argues: “I also put myself in the shoes of the teacher who struggles to understand and discern what is linked to a bilingualism fatigue, adaptation and what instead can be biological of the child himself” (S2).

Given the many requests for certification coming from school contexts, they emphasize, almost with a pungent tone, that the Neuropsychiatry Service “works almost exclusively to give these answers to the school” (S8), to which too many requests arrive that they cannot manage, lengthening the process times too much and strongly reducing meetings with schools, in particular a neuropsychiatrist argues: “Teachers often feel a bit alone and seek our figure for guidance, the problem is that they clog up our service” (S4). Quote S8 reveals a discourse of overload in the neuropsychiatric system, reflecting broader tensions between supply and demand for specialist services in the educational context.

### 3.5 The request for certification to the neuropsychiatry service: the difficulty in sharing

To access a Neuropsychiatry Service, neuropsychiatrists prescribe that there must be a clear and shared agreement between school and families, as the foundation of this process requires strong motivation from parents, considered the sole responsible parties who can request and bureaucratically initiate a certification process. In cases where this mutual understanding is absent, as often occurs with foreign families, the process cannot commence. Specifically, some neuropsychiatrists explain: “There must be a clear and shared agreement between teachers and families” (S5) or “The triage issue is motivation: why do you, as a parent, want to send your child here, not simply to silence the teacher” (S1). Quotes S5 and S1 reveal how the discourse of “sharing” and “agreement” between school and family is constructed as an essential prerequisite for accessing services. This can reflect and reproduce existing power relations in the educational and healthcare systems. The continuation of the evaluation, and especially its utilization, appears to depend on the extent to which the parent can accept its outcome or share its utility. This potential synergy, according to the neuropsychiatrists' perspective, “could prevent the waste of time and resources” (S9), meaning that the evaluation might be interrupted in progress or its outcome may not be adequately utilized by parents. Quote S9 reveals a discourse centered on efficiency and resource use, which could reflect and reproduce neoliberal logics in the healthcare and educational systems. The process can involve several critical aspects; in particular, a neuropsychiatrist explains, “Sometimes it's a clear and shared process, and sometimes it's not” (S3). For instance, it happens that the parent believes only the teacher perceives the problem or, conversely, that their child has a severe problem. The parents' response to this notion can lead to vastly different choices, including relying on Services or failing to recognize what teachers have reported, effectively creating an opposition to the teachers' role. Quote S3 highlights how the “clear and shared process” is discursively constructed as the ideal, potentially marginalizing more complex or ambiguous situations.

Consequently, neuropsychiatrists, teachers, and parents consider the management of communication among various roles as a salient and critical moment. Communication difficulties stem from a “highly bureaucratized and unclear procedure” (T12) that leads to a lack of contact between School and Service, to the extent that some parents offer themselves as facilitators to resolve communication issues between schools and neuropsychiatry services or opt to resort to private neuropsychiatry services. Quote T12 reveals a critical discourse on bureaucracy, constructing it as an obstacle to effective communication. This can reflect tensions between institutional practices and individual needs. Guided by their own theories, we have also observed that teachers often distinguish and judge how different families react to the communication of difficulties to initiate a reporting process, considering some parents as “unconscious” because they fail to understand and accept that their child may have “issues”; in particular, a teacher expresses: “A mother of a girl, understanding her daughter's difficulties, was herself who asked us how to obtain a certification. Unlike another girl who, despite repeated calls we made to the family, the parents remained unconscious from this point of view” (T14). Quote T14 shows how parents are discursively categorized as “aware” or “unaware,” potentially reproducing power and knowledge hierarchies between teachers and parents. In addition to all this, there are difficulties in linguistic comprehension; specifically, a teacher states that families of foreign students “do not believe in the school and would not pursue the reporting” (T22). Quote T22 reveals a discourse that constructs foreign families as “non-believers” in the school system, potentially reproducing cultural stereotypes and marginalizing these families in the educational process.

### 3.6 Ethnographic notes

As per institutional procedure, the request for a diagnostic evaluation must be made by the parent of the minor suspected of having certain academic difficulties. The manner in which referrals are made to Municipal or Neuropsychiatry Services is heavily influenced by how these difficulties are identified by teachers at school and subsequently reported to the parents themselves. Neuropsychiatry Services are generally activated by parents who request an evaluation. The timeframe for such evaluations varies from service to service, ranging from approximately 6 months to about 2 years. Municipal Services intervene by providing consultation with social workers, psychologists, and educators (Iudici and Gagliardo Corsi, 2017; Iudici et al., 2018). The latter are typically activated following the validation of a diagnostic evaluation, while psychologists and social workers are always active within the mandate of Personal Services. From what we observed in the interviews, the procedure does not seem very clear; in some cases, the teacher directly contacts the Neuropsychiatry Service, in others they write a brief referral, and in others, they urge the parent to approach the service either voluntarily or forcibly, that is, under significant pressure. For instance, the terms used to influence the parent are peremptory, and the content concerns the gravity of the situation.

Although good service practice (Consensus) would recommend reporting a difficulty no earlier than 7 years of age (to allow for developmental possibilities), both the interviewed teachers and parents seem driven by interventionist pressure, as if a late referral could compromise development itself.

In many cases, we noticed how discourses on minors' difficulties had the school's demands as an invisible center of gravity. It's as if deviation from the didactic norm opened up suspicion of a psychological problem that would justify its existence.

While it's true that difference from didactic and environmental expectations becomes a criterion for defining a minor in difficulty, we also note that among the discourses presented by all roles involved, there emerges the idea that Italian schools should acculturate you. Attending school has an intrinsic status value that probably focuses attention on performance or level of knowledge rather than on other aspects such as wellbeing, acceptance of diversity, and relationships.

Overall, Neuropsychiatry Services intervene only based on referrals from parents and indirectly from teachers, thus having a role in the final phase of the process leading to certification. Indeed, Neuropsychiatry Services do not directly participate in observing the student before deciding if a potential referral is necessary. While neuropsychiatry services merely prescribe the need for consensus between the family and the group of teachers, the outcome is that there isn't always agreement on what to look for in the minor's developmental process. First and foremost, we must say that there are no upstream regulations on how to manage a minor's difficulty, that is, in the introductory meetings between School and Family, no mention is made of what happens in case of minors' difficulties. This configures the moment of possible referral as an always new situation and does not offer tools to prepare for its management.

## 4 Discussion

Regarding practices for identifying student difficulties in the Italian school context, it clearly emerges that both teachers and parents, in the absence of formalized tools, primarily rely on a comparative approach and their personal theories to recognize potential issues in students. This approach aligns with the presence-participation-success (PPS) model developed by (Ainscow and Miles 2009), which provides a framework for inclusive practices. Comparison with peers emerges as the main method for identifying difficulties. While intuitive, this approach raises questions about its effectiveness and accuracy. As highlighted by (Florian and Black-Hawkins 2011), an excessive focus on developmental norms can lead to neglecting individual diversity in learning and development. Furthermore, this practice could contribute to what (Ainscow 2005) defines as “deterministic thinking,” where difficulties are seen as intrinsic to the student rather than as the result of interaction with the educational environment.

The PPS model, while offering a useful structure for inclusive activities, risks oversimplifying the complexity of educational experiences. As (Slee 2013) argues, this approach may not adequately address systemic barriers to inclusion, focusing primarily on individual aspects of the school experience rather than on the inadequacies of the educational system itself.

The temporal persistence of a problem is often interpreted as an indicator of severity. However, as evidenced by (Kauffman and Landrum 2013), this view could lead to underestimating intermittent or less evident, but equally significant difficulties. This relates to the broader issue of medicalization of diversity, as discussed by (Conrad 2007); (Conrad and Slodden 2012), where the tendency to interpret individual differences through a medical lens has led to a significant increase in diagnoses and clinical interventions in the educational context.

It is interesting to note how consensus among colleagues plays a crucial role in validating a teacher's concerns. While this can offer a broader perspective, it could also lead to a “culture of consensus” that reinforces existing prejudices. This point resonates with the sociocritical perspectives mentioned by (Annamma et al. 2013) and (Artiles 2011), which invite a critical examination of how discursive and institutional practices in schools contribute to creating and maintaining categories of “normality” and “diversity.”

The tendency to give more attention to “evident” or visible difficulties reflects what (Goffman 1963) defined as “visible stigma.” This bias could lead to neglecting less apparent but equally significant problems. As noted by different autjors (Riddick, 2012; Shifrer, 2013; Barg et al., 2010; Gibbs and Elliott, 2015), diagnostic labels, while sometimes useful for accessing specific supports, can also lead to stereotypes and discrimination, negatively influencing students' self-perception and educators' expectations.

This observation aligns with the critique of the reductionist approach inherent in the medicalization of diversity, where human complexity is reduced to simplified diagnostic categories.

The variability in the timing of referrals, with some opting for prompt intervention and others preferring to wait, reflects a broader debate in the field of special education. While authors like (Fuchs and Fuchs 2006) argue for the importance of early intervention, others like (Slee 2011) caution against the risks of excessive medicalization of school difficulties. The active role of parents in the identification process, highlighted in this study, finds support in international literature. However, as noted by (Shifrer 2013), (Barg et al. 2010), and (Gibbs and Elliott 2015), diagnostic labels, while sometimes useful for accessing specific supports, can also lead to stereotypes and discrimination, negatively influencing students' self-perception and educators' expectations. (Epstein 2010) emphasizes the importance of an effective partnership between school and family for students' educational success.

A clear contrast emerges between the approach adopted by families and that of the school regarding how to approach the first signs of difficulty. Families tend to experiment with more flexible and personalized strategies, adapting to the specific needs of the child. This more individualized approach seems to lead to better modulation of the child's behavior in the domestic environment. On the other hand, the school context appears more rigid, with teachers evaluating students' difficulties primarily based on their adherence to school norms and ministerial requirements.

This discrepancy reflects a broader debate in the field of education, between inclusive approaches and more traditional methods. As highlighted by (Ainscow et al. 2006), inclusion requires a profound transformation of school culture, which goes beyond the simple adoption of specific teaching strategies.

The strong emphasis on standards and ministerial requirements by teachers suggests a certain rigidity in the Italian school system. This approach could limit the ability to respond effectively to the diverse needs of students, as evidenced by (Florian and Black-Hawkins 2011) in their study on inclusive pedagogy. The tendency to focus primarily on didactic and performance aspects, neglecting relational and social ones, reflects a narrow view of the student's role. This contrasts with the holistic approach to education proposed by authors such as (Noddings 2013), which emphasizes the importance of relationships and emotional wellbeing in learning. The child neuropsychiatrist's comment on teachers' failure to consider “neurodiversity” highlights the need for greater collaboration and communication between the educational and health sectors. This aspect is particularly relevant in the Italian context, as highlighted by (Ianes 2006) in his work on school integration. The intervention strategies adopted by teachers seem to focus mainly on cognitive and didactic aspects, such as personalized explanations or the use of dispensative and compensatory tools. However, this approach might neglect other important dimensions of learning, as suggested by (Gardner 1983) in his theory of multiple intelligences. The tendency to consider “pathological” behavior that does not adhere to school norms raises concerns about the possible medicalization of school difficulties. This theme has been widely discussed by authors such as (Slee 2013), who warn against the risks of excessive pathologization of individual differences in the educational context.

The analysis of the results also reveals a significant trend in the Italian school context: the concept of student “discomfort” is often interpreted as a failure to adhere to the norms and expectations of the school environment (Iudici and Fabbri, 2017). This perspective, shared by both teachers and parents, raises important questions about the perception and management of diversity in the education system.

The interviewed teachers show particular attention to behaviors that do not conform to school rules, such as difficulty staying seated, lack of respect for coexistence norms, or problems in interacting with peers. This focus on behavior as an indicator of discomfort reflects a view that could be considered traditional of learning and classroom behavior. This approach finds support in international studies, such as that of (Thornberg 2010), which highlighted how school rules and behavioral expectations can significantly influence the perception of student discomfort.

On the other hand, this perspective could lead to a potential pathologization of behaviors that simply do not align with pre-established school norms. As highlighted by (Armstrong 2012), there is a risk of confusing behavioral or learning diversity with actual situations of discomfort, leading to potentially inappropriate or tizing interventions. Parents seem to adopt criteria similar to those of teachers in recognizing their children's difficulties, often expressing a sense of unmet expectations regarding “appropriate” behavior for age. This alignment between teachers' and parents' perceptions could indicate a culturally shared vision of what constitutes “normal” or “acceptable” behavior in the school setting (Iudici et al., 2021).

However, it is important to note that this approach might neglect other important indicators of student wellbeing or discomfort. As highlighted by (Cefai and Cooper 2010) in their study on students with emotional and behavioral difficulties, it is fundamental to consider the perspectives of the students themselves to fully understand their experiences and needs.

Furthermore, the strong emphasis on “sitting still” and respecting rules raises questions about how prepared the school system is to accommodate different modes of learning and expression. Studies such as that of (Noddings 2013) suggest the importance of a more inclusive and flexible approach to education, which considers diversity as a resource rather than a problem to be solved. But on this, it is necessary for schools to be structurally able to implement it.

From the responses, a tendency emerges to delegate responsibility to external experts when teachers and parents are faced with behaviors that do not correspond to societal norms and expectations. This practice reflects what (Skrtic 1991) has defined as “bureaucratic professionalism” in special education. However, such an approach can lead to excessive medicalization of school difficulties, as highlighted by (Slee 2011) in his work on inclusive education. Another critical aspect concerns teacher preparation. The criticisms raised by neuropsychiatrists about teachers' ability to correctly complete observation grids highlight a significant training gap. This calls attention to the importance of teacher training for the effective implementation of inclusive practices, as emphasized by (Avramidis and Norwich 2002). The reported texts also highlight the growing pressure on neuropsychiatry services, which find themselves “clogged” with requests from schools. This situation reflects a broader trend toward the medicalization of education, a phenomenon discussed by (Conrad 2007) in his work on the medicalization of society.

Another critical point concerns the lack of direct communication between schools and neuropsychiatry services before formal referral. This practice contrasts with international best practices which, as highlighted by (Ainscow et al. 2006), emphasize the importance of multi-agency collaboration for effective inclusive education. Family involvement in the decision-making process, although present, often seems to be guided, in many cases influenced, by the school. This aspect calls attention to the importance of a true school-family partnership (Iudici, 2015). A particularly concerning element is the absence of clear regulations on how to manage student difficulties. This systemic gap contrasts with the recommendations of (Booth and Ainscow 2011), who in their Index for Inclusion emphasize the importance of clear policies and practices to promote inclusion and health (Turchi et al., 2022).

Finally, the search for a diagnosis as a “form of help” raises concerns about student labeling. (Florian and Black-Hawkins 2011) warn against the risks of a deterministic approach to learning difficulties, which could limit students' expectations and opportunities.

A central aspect that emerges with all its force is the need for a clear and shared agreement between school and family to initiate the certification process with neuropsychiatry services. This emphasis on collaboration recalls the concept of “educational partnership” discussed by (Epstein 2010) in her model of parental involvement. However, the texts detected by the research highlight how this collaboration is often difficult to achieve, especially with families of foreign origin, revealing potential cultural and linguistic barriers in accessing services. This aspect reflects the challenges identified by (Hornby and Lafaele 2011) in their work on barriers to parental involvement in education. The authors emphasize how differences in perception between teachers and parents regarding student difficulties can hinder effective collaboration. Some responses from neuropsychiatrists clearly revealed an accommodating approach by parents to the certification process, which is also present in literature, also called a tendency toward “bureaucratization” of the reporting process, which can create further obstacles to effective communication. This observation aligns with the criticisms raised by (Slee 2011) of educational systems that tend to medicalize and bureaucratize responses to student difficulties, potentially at the expense of more inclusive and flexible approaches.

Another interesting aspect is the active role that some parents assume as “facilitators” between school and neuropsychiatry services. This phenomenon draws attention to the importance of parental involvement in the educational process, particularly with regard to expectations (Turnbull and Turnbull, 2015; Haines et al., 2015). The distinction made by some teachers between “aware” and “unconscious” parents raises significant ethical issues. This categorization could reflect cultural and social prejudices, which can occur toward families from other cultural contexts (Harry, 2008).

Finally, the specific difficulties encountered with families of foreign students highlight the need for culturally sensitive approaches in inclusive education (Banks, 2020).

This study highlights the complexity of the process of reporting and certifying student difficulties in the Italian context. The need to develop more effective strategies for communication and collaboration between school, family, and services clearly emerges, taking into account the various cultural, linguistic, and social barriers. As suggested by (Ainscow et al. 2006), a truly inclusive approach requires a systemic rethinking of educational practices and relationships among all actors involved in the educational process.

## 5 Conclusion

In order to understand the reasons that lead to requests for multiple certifications, in this study we highlighted the criteria and social discourses that teachers, parents and services use when reporting a difficulty that could lead to certification. The research reveals the absence of a formalized protocol for defining when and how reporting should take place, reflecting the complexity and contradictions in the processes of constructing diversity in schools.

However, there is a shared practice of making collective decisions in class councils. In some cases, this step seemed more of a formality than a substantive act, making it a place for agreement rather than a context for discussing criteria.

A salient feature that emerges from the analysis is the tendency to interpret pupils' difficulties primarily as a deviation from the expectations and norms of the school environment. This paradigm, although seemingly logical, raises crucial questions about the perception of and approach to diversity in the educational context. The focus on unconventional behavior as indicators of distress reflects a traditional conception of learning, which risks labeling as problematic manifestations that may simply represent variations in normal development and individual expression.

A critical aspect highlighted by the research is the tendency to delegate responsibility to specialists, a phenomenon that could reinforce what some authors call the psychologisation or psychiatrisation of normality (Conrad, 2007; Frances, 2010). This process also conceals the idea that without the confirmation of a specialist, teachers and parents cannot continue because they develop the idea that they are counterproductive or harmful. This leads to two reflections. The first is that the child seems to be at an identity crossroads: student or person with a diagnosis? The second concerns the fact that timely (or preventive) intervention may even compromise developmental progress, as also found by (Slee 2013). This inclination leads to excessive demand for help from neuropsychiatric services, which are unable to provide a comprehensive response. For example, it has been noted that the time spent by these services is more focused on diagnosis than on observation at school or even direct collaboration with teachers.

Another noteworthy element is the discrepancy between the flexible and personalized approach and the rigidity of the school system, which is anchored to ministerial standards and requirements. This divergence reflects a broader debate in the field of education between inclusive methodologies and more conventional approaches. The survey highlights significant shortcomings in communication between schools, families and specialist services. These gaps hinder the implementation of effective inclusive practices and the creation of genuine educational synergy between all actors involved.

The research also highlights the need for a clear and shared agreement between schools and families in the certification process, emphasizing the importance of a true educational partnership. However, significant challenges emerge in achieving this collaboration, especially with families of foreign origin, revealing cultural and linguistic barriers to accessing services.

The results of this exploratory research highlight the urgent need for a systemic reconsideration of educational practices and relationships between all those involved in the educational process. It is imperative to develop more inclusive and adaptable approaches that value diversity, including cultural diversity, as a resource rather than perceiving it as an obstacle to be overcome. This paradigm shift requires not only a review of teaching methodologies, but a more profound transformation of school culture and educational policies. Future developments in this field should aim to integrate the insights of different approaches, promoting an educational model that genuinely values diversity as a resource rather than as a problem to be solved. Only through a critical and multidimensional understanding of the processes of constructing diversity can we hope to create truly inclusive and equitable educational systems for all students.

The implications of this research suggest the need for targeted interventions on several fronts: strengthening teacher training, with a particular focus on inclusive skills; improving communication channels between schools, families and specialist services; developing clear and flexible protocols for managing student difficulties; and promoting a culturally sensitive approach to inclusive education, especially in multicultural contexts. This work paves the way for further exploration and reflection, inviting constructive dialogue between educational theory and practice with a view to creating a truly inclusive school system that responds to the diverse needs of all students, who would benefit from being involved in the process of reorganizing inclusive practices.

Research limitations: a limitation of the research concerns the fact that the study was conducted in two different regions, thus the results should be understood in terms of exploration and not generalization on a national level. This is also due to the snowball sampling effect, which may not be representative of the broader population of teachers, parents, and healthcare and municipal operators. The data might not reflect the reality of other regions. For the same reasons, participants in snowball sampling might be very homogeneous, with participants sharing similar characteristics or opinions (Noy, 2008). A further limitation may be related to the fact that participants who volunteer might have different characteristics or opinions from those who do not participate, introducing a potential bias in this case as well (Sedgwick, 2013). Finally, another possible bias could be related to gender, as there is a clear gender disparity in the group of teachers (20 females vs. 8 males), which could influence the results. However, this proportion falls within the generality of Italian schools.

## Data Availability

The original contributions presented in the study are included in the article/supplementary material, further inquiries can be directed to the corresponding author.
